# Immortalised canine buccal epithelial cells’ CXCL8 secretion is affected by allergen extracts, Toll-like receptor ligands, IL-17A and calcitriol

**DOI:** 10.1186/s13567-022-01090-5

**Published:** 2022-09-13

**Authors:** Michael Pelst, Clara Höbart, Hilde de Rooster, Bert Devriendt, Eric Cox

**Affiliations:** 1 Laboratory of Immunology, Department of Translational Physiology, Infectiology and Public Health, Faculty of Veterinary Medicine, Ghent University, Merelbeke, Belgium; 2 Center of Physiology and Pharmacology, Medical University of Vienna, Vienna, Austria; 3 Small Animal Department, Faculty of Veterinary Medicine, Ghent University, Merelbeke, Belgium

**Keywords:** Buccal, epithelial cell, CXCL8, calcitriol, oral, canine

## Abstract

Epithelial cells are known to produce mediators which can influence the behaviour of neighbouring immune cells. Although the oral mucosa has gained increased interest as a route to induce allergy desensitisation and mucosal pathogen immunisation in dogs, there is only limited knowledge on the factors which impact mediator secretion by canine oral epithelial cells. The study’s objective was to enlarge the knowledge on the stimuli that can influence the secretion of some pro- and anti-inflammatory cytokines and the chemokine CXCL8 by canine buccal epithelial cells. To investigate this, buccal epithelial cells were isolated from a biopsy of a dog and immortalised by lentiviral transduction of the SV40 large T antigen. The cells were stained with a CD49f and cytokeratin 3 antibody to confirm their epithelial origin. Cells were incubated with allergen extracts, Toll-like receptor ligands (TLRL), recombinant cytokines and vitamin A and D metabolites. Subsequently, the secretion of the cytokines interleukin (IL)-4, IL-6, IL-10, IL-17A, IFN-γ, TGF-β1 and the chemokine CXCL8 was assayed by ELISA. Immortalised canine buccal epithelial cells stained positive for CD49f but not for cytokeratin 3. The cells produced detectable amounts of CXCL8 and TGF-β1. A *Dermatophagoides farinae* extract, an *Alternaria alternata* extract, Pam3CSK4, heat-killed *Listeria monocytogenes*, FSL-1, flagellin and canine recombinant IL-17A significantly increased CXCL8 secretion, while the vitamin D metabolite calcitriol significantly suppressed the production of this chemokine. This study showed that certain allergens, TLRL, IL-17A and calcitriol modulate CXCL8 secretion in a cell line of canine buccal epithelial cells.

## Introduction

The oral mucosa is an immunologically active tissue capable of inducing tolerance for antigens. In humans and mice, this tolerizing role can be partly attributed to the lack of local pro-inflammatory effector cells present, with only limited numbers of mast cells and eosinophils residing in oral tissues [1, 2]. The anti-inflammatory properties of the oral mucosa likely impair the maturation process of local dendritic cells which alters these cells’ surface marker expression, subsequently having a beneficial effect on the tolerizing responses at the T cell level [3–5]. Because of this, administration of allergens in the oral cavity is used to desensitise humans and dogs to environmental and food allergens [6–9]. The allergens could impact the secretion of mediators by the cells they encouter. In dogs this has been seen for house dust mite allergens and skin epithelial cells [10, 11], but the effect of allergens on canine buccal epithelial cells has not yet been studied. Apart from antigen-specific tolerance, also pathogen-specific protective mucosal immune responses can be generated by vaccines that are administered onto the oral mucosa [12–14]. Recently, an oral *Bordetella bronchiseptica* vaccine has been developed for dogs which involves dripping the vaccine antigens onto the oral epithelium [15]. In the future, such vaccines could contain adjuvants, such as Toll-like receptor ligands (TLRL) and vitamin metabolites, to enhance the immune response [16, 17].

During oral immunisations, the antigen is taken up and processed by the oral mucosal immune system after an initial contact with the oral epithelium [12, 18]. Although oral epithelial cells are the first to come in contact with the antigen, there is only limited knowledge on factors which influence these cells’ innate immune activity. Yet, epithelial cells are capable of producing mediators which can strongly influence the outcome of immune responses [19, 20]. Epithelial cells express amongst others receptors for cytokines, pathogen-associated molecular patterns and vitamins. Activation of these receptors can trigger the release of specific mediators such as thymic stromal lymphopoietin (TSLP) and CXCL8 [21–23], which, in the skin, are known to propagate a pro-allergic and pro-inflammatory immune response, respectively [24–27]. However, also cytokines secreted by cells in close vicinity, can influence the secretion of pro- and anti-inflammatory mediators by epithelial cells. Indeed interferon-γ (IFN-γ) and interleukin (IL) 17A have been shown to trigger the release of pro-inflammatory mediators by canine skin epithelial cells [22, 28]. Whether cytokines can influence mediator secretion by canine buccal epithelial cells has not been investigated yet.

The limited knowledge on the immune function of oral epithelial cells might be attributed to the fact that primary epithelial cells can only be cultured in vitro for a short period of time due to cell senescence. This limits the amount of data that can be obtained of cells isolated from a single biopsy. Fortunately, the increasing knowledge on cellular immortalisation techniques provides the opportunity to study epithelial cells through generation of a long-term to infinite culture of a single epithelial cell type [29]. To generate immortalised epithelial cell lines from a biopsy, the epithelial cells first have to be separated from other cell types present in the biopsy. To confirm the epithelial origin of these cells, stainings for intra- and extracellular markers can be performed. In dogs, it was shown that the surface marker CD49f can be used to identify skin epithelial cells and primary sublingual epithelial cells [30, 31]. In humans, intracellular cytokeratin 3 was shown to be present in cultured buccal epithelial cells [32].

To perform allergy desensitisation through the oral mucosal immune system, allergens are often administered onto the sublingual mucosa [8]. Still, also the buccal mucosa has been proposed as an interesting entry point to deliver antigens in the oral cavity. From one perspective, the use of the sublingual mucosa as a route for antigen delivery seems more feasible, since the thicker buccal epithelium limits the passage of allergens into the lower epithelial cell layers where the antigen-presenting cells (APCs) reside. On the other hand, the sublingual surface is exposed to a stronger salivary wash-out than the buccal mucosa, shortening the duration that antigens can be taken up by the APCs. Interestingly, in humans, the buccal epithelium has a higher density of Langerhans-like dendritic cells than the sublingual mucosa, which likely increases the chance that antigens are collected buccally by these APCs [1, 18].

This study investigated whether canine buccal epithelial cells could be immortalised by transduction of the simian virus 40 (SV40) large T antigen and whether exposure of these cells to allergen extracts, TLR-Ligands, recombinant cytokines and vitamin metabolites could influence their secretion of inflammatory molecules.

## Materials and methods

### Immunohistochemistry on cryosections of a buccal biopsy of a dog and a pig

To identify which markers can be used to stain epithelial cells of the canine buccal mucosa, cryosections of the canine and porcine tissue were stained with a CD49f- and cytokeratin 3-specific antibody. Buccal biopsies of approximately 1 × 1 cm and 0.5 cm in depth collected from the center of the inner surface of the cheek were obtained from the corpse of a dog and a pig. The sample of the pig was included in the study as a positive control for the characterisation of the epithelial cells since previous experiments by our lab (research not published) had shown that epithelial cells of these oral biopsies stain positive for both cytokeratin 3 and CD49f. The pig was euthanized for experimental purposes other than the collection of this biopsy. The dog (White Swiss Shepherd, 9 years) was euthanized for medical reasons (neurological disease, severe ataxia, no definitive diagnosis) unrelated to this study. Oral consent was given by the owner to use the dog’s corpse for scientific purposes. Biopsies were collected within 3 h after euthanasia. The samples were submerged in methocel (Merck, Burlington, MA, USA), snap-frozen in liquid nitrogen and stored at −80 °C. Tissue blocks were sectioned at 10 µm thickness (LEICA CM3050 S Microtome, Leica, Wetzlar, Germany), applied onto APES-coated glass slides and submerged in acetone for 10 min at −20 °C. Subsequently, slides were washed (PBS + 1% BSA, Merck) followed by 30 min blocking at room temperature (RT; 18–22 °C) with PBS + 1% BSA + 5% goat serum (Merck). After a washing step, slides were incubated with the CD49f or cytokeratin 3/2p antibody or the isotype control (Table 1) for 1 h at RT followed by washing. Secondary antibody (Table 1) was added to all slides for 1 h at RT followed by washing and slides were counterstained with Hoechst (Thermofisher Scientific, Waltham, MA, USA), followed by 3 washing steps with PBS. Slides were analysed using a Leica Leitz DMR fluorescence microscope (Leica). Images were processed using ImageJ [33].

**Table 1:** **Antibodies used for immunohistochemistry and flow cytometry**

	Antibody	Clone	Working concentration	Supplier
Primary antibody	Rat anti-human CD49f	NKI-GoH3	10 µg/mL	Thermofisher Scientific, Waltham, MA, USA
	Mouse anti-rabbit cytokeratin 3/2p	AE5	10 µg/mL	Santa Cruz Biotechnology, Dallas, TX, USA
Isotype control	Rat IgG2a anti-KLH	RTK2758	10 µg/mL	Biolegend, San Diego, CA, USA
	Mouse IgG1 anti-F4	IMM01	10 µg/mL	In-house hybridoma
Secondary antibody	FITC-conjugated goat anti-mouse IgG	Polyclonal	5 µg/mL	Biolegend, San Diego, CA, USA
	FITC-conjugated goat anti-rat IgG	Polyclonal	5 µg/mL	Biolegend, San Diego, CA, USA

### Isolation of buccal epithelial cells from a dog

To isolate canine buccal epithelial cells, a buccal biopsy of approximately 1 × 1 cm and 0.5 cm in depth collected from the center of the inner cheek was obtained from another privately-owned dog (Labrador Retriever, 5 years) that was euthanised for medical reasons (discus hernia) unrelated to the study, within 3 h after euthanasia. Also for this dog, oral consent was given by the owner to use the dog’s corpse for scientific purposes. Periodontal disease was not checked prior to the biopsy. No histopathological analysis was performed on the biopsy. The biopsy was briefly submerged in 70% ethanol, washed 3 times with Ca^2+^- and Mg^2+^-free Dulbecco’s PBS (DPBS, Thermofisher Scientific) and incubated in 4 mg/mL Dispase II (Thermofisher Scientific) in DPBS on ice. After 24 h incubation, the epithelial layer was carefully separated from the underlying subepithelial tissue using two sterile forceps, followed by incubation of the epithelial tissue in DPBS with 0.25% trypsin (Thermofisher Scientific) and 2.65 mM EDTA (VWR, Radnor, PA, USA) for 20 min at RT. Subsequently, the cell suspension was resuspended, trypsin was neutralised with an equal volume of 5% fetal calf serum (FCS) (Merck) in DPBS and the cells were filtered over a 70 µm cell strainer (Merck). After centrifugation (400 *g*, 10 min, 18 °C), the cells were counted using a hemocytometer.

### Immortalisation of canine buccal epithelial cells

To immortalise the cells, the primary buccal epithelial cells were seeded at 10 000 cells/cm^2^ in a 6-well plate in epithelial cell culture medium (ECCM) and incubated (37 °C, 5% CO_2_) for 24 h. Supernatant was removed and 2 mL of SV40T Lentivirus viral supernatant (Applied Biological Materials, Richmond, Canada) was added with 10 µg/mL polybrene (Applied Biological Materials) overnight. Subsequently, viral supernatant was removed and replaced by ECCM. ECCM consisted of ¾ DMEM, ¼ nutrient mixture F12-Ham (Thermofisher Scientific), 5% FCS, 2 nM 3,3′,5-triiodo-l-thyronine sodium salt, 5 µg/mL recombinant human insulin, 10 ng/mL recombinant human epidermal growth factor, 0.4 µg/mL hydrocortisone, 100 nM l-isoproterenol hydrochloride (Merck), 100 U/mL penicillin, 100 µg/mL streptomycin and 100 µg/mL gentamicin (Thermofisher Scientific) [32, 34].

### Detection of SV40T transduction by PCR

To assess whether the incubation with the SV40T lentivirus had transduced the SV40 large T antigen sequence in the canine buccal epithelial cells, a PCR analysis was performed according to the following procedure: (1) DNA was extracted from cells that were cultured with or without the SV40T Lentivirus viral supernatant using the QIAamp DNA Mini and Blood Mini Kit according to the manufacturer’s instructions (Qiagen, Hilden, Germany), (2) the PCR reaction was performed in a PCR buffer containing 200 µM PCR grade nucleotide mix, 0.05 U/µL Faststart Taq polymerase (FastStart™ Taq DNA Polymerase, dNTPack, Merck), 300 nM SV40 large T antigen forward (AGCCTGTAGAACCAAACATT) and reverse primer (CTGCTGACTCTCAACATTCT) and 20 ng/µL DNA, and PCR cycling consisted of an initial heating reaction at 95 °C for 4 min followed by 40 cycles of denaturation at 95 °C (30 s), annealing at 52 °C (30 s) and elongation at 72 °C (60 s), (3) the PCR products were resuspended in loading buffer (30% glycerol, 0.25% bromophenol blue) and loaded onto a 1% agarose gel containing 0.01% GelRed followed by gel electrophoresis for 30 min at 100 V. An image of the bands was captured using a Gel Doc EZ (Bio-Rad, Hercules, CA, USA).

### Determination of CD49f expression by immortalised canine buccal epithelial cells using flow cytometry

To assess whether the immortalised cells were of epithelial origin, the expression of CD49f was analysed by flow cytometry. Fifty thousand immortalised canine buccal epithelial cells were added to conical bottomed wells of a 96-well plate. Details on the used isotype control and CD49f antibody are shown in Table 1. Primary antibodies and corresponding isotype controls were added in a volume of 50 µL PBS + 1% BSA for 20 min on ice. Washing of the cells was followed by addition of 0.6 µg/mL Alexa Fluor® 647 goat anti-rat IgG in 50 µL for 20 min on ice. After two washing steps, cells were resuspended in 100 µL DPBS and the plate was measured using a CytoFLEX flow cytometer (Beckman Coulter, Brea, CA, USA). The results were analysed using the CytExpert 2.0 software (Beckman Coulter), singlet cells were gated in a forward scatter area and height plot, followed by discrimination of cell debris in a forward and side scatter plot. Washing involved adding 100 µL PBS + 1% BSA to each well followed by 3 min centrifugation at 600 *g*.

### Incubation of immortalised canine buccal epithelial cells with allergen extracts, Toll-like receptor ligands, recombinant cytokines and vitamin A and D metabolites

To evaluate the immunological response of the immortalised buccal epithelial cells on exposure to different stimuli with a potential immune-modulating effect, the following procedure was followed: (1) the immortalised canine buccal epithelial cells were seeded in ECCM in a 24-well plate at 30 000 cells/well and incubated overnight at 37 °C in a 5% CO_2_ incubator, (2) 16 h later, allergen extracts, Toll-like receptor ligands, recombinant cytokines and vitamin A and D metabolites were added to the cultured cells. An overview of the used products and concentrations is shown in Table 2. All products were added to the immortalised buccal epithelial cells in a volume of 400 µL epithelial cell stimulation medium (ECSM) (¾ DMEM, ¼ Ham’s F-12, 100 U/mL penicillin, 100 µg/mL streptomycin and 100 µg/mL gentamicin). After 24 h incubation, the supernatant was collected, spun down at 400 *g* for 10 min to remove detached cells and frozen at −20 °C.

**Table 2:** **Products used to stimulate the immortalised buccal epithelial cells**

Group of products	Name	Working concentration	Supplier
Allergen extracts	*Dermatophagoides farinae* (XPB81D3A2.5, Lot: 307,244)	20 µg/mL (2.1 µg/mL Der f 1)	Greer laboratories, Lenoir, NC, USA
	*Dermatophagoides pteronyssinus* (XPB82D3A2.5, Lot: 346,230)	20 µg/mL (0.58 µg/mL Der p 1)	
	*Alternaria alternata* (XPM1D3A2.5, Lot: 312,142)	20 µg/mL	
	Birch (*Betula pendula*) (XP527D3A2.5, Lot: 350,600)	20 µg/mL	
	Timothy grass (*Phleum pratense*) (XP28D3A2.5, Lot: 305,467)	20 µg/mL	
TLR ligands	Heat-killed *Listeria monocytogenes* (HKLM) (TLR2)	10^8^ cells/mL	Invivogen, San Diego, CA, USA
	Pam3CSK4 (TLR1/2)	1 µg/mL	
	FSL-1 (TLR2/6)	1 µg/mL	
	Polyinosinic:polycytidylic acid high molecular weight (poly I:C HMW) (TLR3)	10 µg/mL	
	Polyinosinic:polycytidylic acid low molecular weight (poly I:C LMW) (TLR3)	10 µg/mL	
	LPS (*Escherichia coli* K12) (TLR4)	10 µg/mL	
	Flagellin (*Salmonella* Typhimurium) (TLR5)	1 µg/mL	
	Imiquimod (TLR7)	10 µg/mL	
	ssRNA 40 (TLR8)	10 µg/mL	
	ODN 2006 (TLR9)	5 µM	
Recombinant cytokines	Canine IFN-γ	10 µg/mL	R&D Systems, Minneapolis, MN, USA
	Canine IL-10	10 µg/mL	
	Canine IL-17A	10 µg/mL	
	Canine IL-4	10 µg/mL	
	Canine IL-6	10 µg/mL	
	Human TGF-β1	10 µg/mL	
Vitamins	Calcitriol	1 µM	Merck, Burlington, MA, USA
	All-trans-retinoic acid	10 µM	
	9-cis-retinoic acid	10 µM	

If the product is an agonist of a specific receptor, the receptor is underlined. TLR = Toll-like receptor.

### Cytokine enzyme-linked immunosorbent assays

To assess the effect of the different stimuli on cytokine secretion by the immortalised canine buccal epithelial cells, ELISAs for canine IL-4, IL-6, IL-10, IL-17A, IFN-γ, IL-8/CXCL8 and human TGF-β1 were performed on the cell culture supernatants using the DuoSet ELISA kits (R&D systems, Minneapolis, MN, USA) according to the manufacturer’s instructions. As human and canine TGF-β1 were shown to have high homology [35], human TGF-β1 ELISAs were used to detect canine TGF-β1 [36].

### Statistical analysis

Statistical analysis was performed using R [37]. Normal distribution of the data was assessed and confirmed using the Shapiro–Wilk’s test and quantile–quantile plot. To compare the effect of allergen extracts, TLRL, recombinant cytokines and vitamin A and D metabolites on the cytokine production of immortalised canine buccal epithelial cells, a paired t-test was used. A *p*-value lower than 0.05 was considered significant.

## Results

### CD49f expression and immortalisation of canine buccal epithelial cells

**Figure 1:**
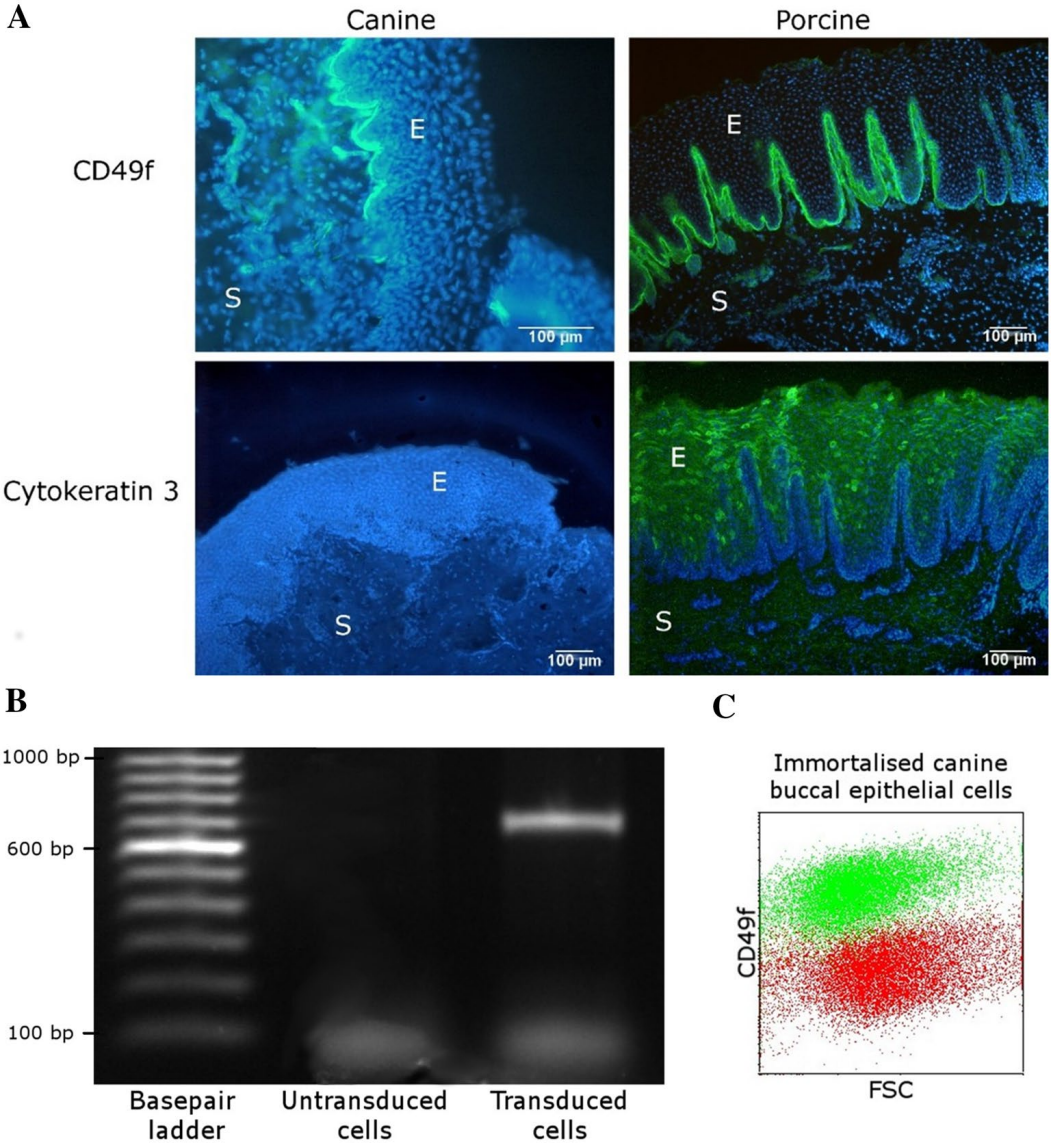
**CD49f expression and immortalisation of canine buccal epithelial cells. A** CD49f and cytokeratin 3 staining of canine and porcine buccal cryosections. A CD49f antibody stains the basal side of the epithelium of the canine and porcine buccal mucosa while a cytokeratin 3 antibody only stains the porcine epithelium. Cryosections of the canine and porcine buccal mucosa were stained with CD49f or cytokeratin 3 (FITC) and counterstained with Hoechst. E: epithelium, S: subepithelial tissue. **B.** The SV40 large T antigen sequence in the genome of canine buccal epithelial cells. DNA extraction, PCR and agarose gel electrophoresis was performed on SV40 large T antigen-transduced and -untransduced canine buccal epithelial cells using an SV40T-specific primer pair. **C.** Immortalised canine buccal epithelial cells express CD49f. Immortalised canine buccal epithelial cells were stained for CD49f (green) or isotype control (red) and analysed by flow cytometry. FSC: forward scatter.

In both theanine and porcine buccal biopsies, the basal layer of the buccal mucosa stained positive for CD49f, while cytokeratin 3 was only detected in the porcine epithelium (Figure 1A). In the extracted DNA of the canine buccal epithelial cells which were incubated with the SV40T lentivirus, a band of 792 base pairs was detected, while amplified DNA of canine buccal epithelial cells cultured without the SV40T lentivirus did not show this band (Figure 1B) demonstrating successful transduction of the cells. Using flow cytometry, it was shown that in vitro-cultured immortalised canine buccal epithelial cells expressed the surface marker CD49f (Figure 1C).

### Immortalised canine buccal epithelial cells increase CXCL8 secretion when exposed to allergen extracts, TLRL, and IL-17A while calcitriol decreases CXCL8 secretion

**Figure 2:**
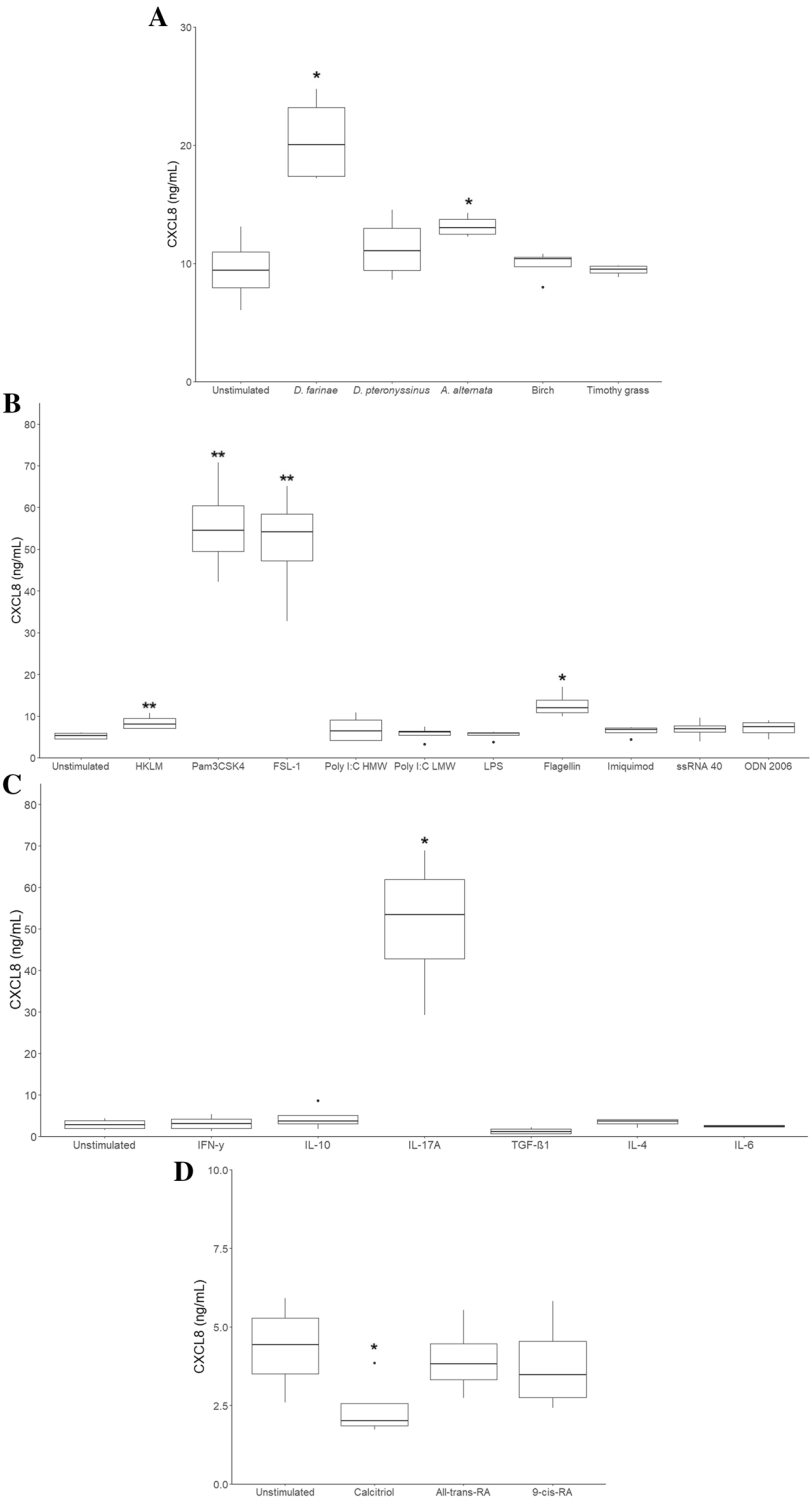
**The effect of allergen extracts, Toll-like receptor ligands, recombinant cytokines and vitamins on CXCL8 secretion by immortalised canine buccal epithelial cells.** Immortalised canine buccal epithelial cells (*n* = 4 experimental days) were incubated for 24 h with a *D. farinae*, *D. pteronyssinus*, *A. alternata*, birch and timothy grass extract (**A**), with HKLM, Pam3CSK4, FSL-1, Poly I:C high (HMW) and low molecular weight (LMW), LPS, flagellin, imiquimod, ssRNA 40, ODN 2006 (**B**), with IFN-γ, IL-10, IL-17A, TGF-β1, IL-4, IL-6 (**C**) and with 1 µM calcitriol, 10 µM all-trans-retinoic acid (all-trans-RA) and 10 µM 9-cis-retinoic acid (9-cis-RA) (**D**). CXCL8 secretion was measured by ELISA. *p < 0.05 to control; **p < 0.01 to control; paired Student’s *t*-test.

Of all 5 allergen extracts to which the immortalised buccal epithelial cells were exposed, only the *Dermatophagoides farinae* and *Alternaria alternata* extracts significantly increased CXCL8 secretion (Figure 2A). Additionally, the production of this chemokine was significantly augmented by the TLR2 ligands (HKLM, Pam3CSK4, FSL-1) and the TLR5 ligand flagellin (Figure 2B), but not by ligands for TLR3, TLR4, TLR 7, TLR8 and TLR9 (Table 2). Recombinant canine IL-17A (Figure 2C) increased CXCL8, but not recombinant canine IL-4, IL-6, IL-10, or IFN-γ. Calcitriol (1,25-dihydroxyvitamin D3) on the other hand significantly reduced the secretion of this chemokine compared to unstimulated cells (Figure 2D), whereas all-trans-retinoic acid and 9-cis-retinoic acid did not affect CXCL8 secretion. Apart from CXCL8, another cytokine detectable in all conditions was TGF-β1. However, the secretion of this cytokine could not be affected by any of the tested stimuli (data not shown). Interleukin-4, IL-6, IL-10, IL-17A or IFN-γ were undetectable by ELISA in the supernatant of the immortalised canine buccal epithelial cells.

## Discussion

To the authors’ knowledge, this is the first study which characterised CD49f expression and cytokine secretion by canine buccal epithelial cells. CD49f was shown to be expressed at the basal side of the buccal epithelium in tissue sections and, when cultured in vitro, this marker was still detected on immortalised canine buccal epithelial cells by flow cytometry. These results further imply that the surface marker CD49f is commonly expressed by epithelial cells that originate from the basal layer. After all, human oral epithelial cells [38] and canine epidermal keratinocytes [30] cultured in vitro also showed constitutive expression of this marker. Additionally, in the human epidermis, basal epithelial cells were shown to express CD49f [39, 40] and also in dogs, the dermal–epidermal junction of footpad tissue stained positive for CD49f [41]. Interestingly, the antibody used to detect cytokeratin 3 did not stain the epithelium of the canine buccal tissue while this marker does stain the oral epithelium of pigs and of humans [32]. The oral mucosa of dogs might therefore not express cytokeratin 3 or dogs might express a variant of this filament which cannot be recognised by the used antibody. Still, the percent identity between the human and dog cytokeratin 3 protein sequences is 83.09% [42].

In order to perform a multitude of experiments with buccal epithelial cells obtained from a single biopsy, the cells were immortalised by transduction of the SV40 large T antigen. This large T antigen binds to the heat shock chaperone hsc70, to p53 and proteins of the retinoblastoma family [43], transformations which promote limitless cell division but can also impact the cells’ mediator secretion [44]. This is a limitation of the study, the findings must therefore be interpreted with caution and should be confirmed by experiments using primary buccal epithelial cells.

Upon the immortalisation, production of IL-4, IL-6, IL-10, IL-17A, IFN-γ, CXCL8 and TGF-β1 was analysed by ELISA. We observed that the cells only secreted the pro-inflammatory chemokine CXCL8 and the cytokine TGF-β1. Human oral epithelial cells also express these two mediators as well as the pro-inflammatory cytokine IL-6 [45]. Canine epidermal keratinocytes are potent producers of CXCL8 and these cells also produce IL-10 and IFN-γ [46, 47]. Interestingly, production of IL-6, IL-10 or IFN-γ was not detected for the immortalised buccal epithelial cells. It should be investigated whether this low variety in detectable mediators is due to specific properties of canine buccal epithelial cells. CXCL8 is a pro-inflammatory chemokine which is capable of attracting neutrophils and T lymphocytes [27, 48]. Recently, single-cell sequencing of human buccal and gingival epithelial cells also showed that genes of the leukocyte chemotaxis pathway, such as CXCL8, were strongly expressed [49].

The main objective of this study was to get a better understanding of which substances are able to influence buccal epithelial cells’ cytokine and chemokine secretion. To this end, the response to TLRL, cytokines, vitamin metabolites and allergens was analysed. Application of allergens onto the oral epithelium is practised to desensitise allergic individuals. Allergen extracts are able to interact with receptors on epithelial cells, influencing the cells’ mediator secretion [50]. In this study an extract of *D. farinae* and *A. alternata* significantly upregulated CXCL8 secretion by the immortalised buccal cells. In our previous study, *D. farinae* extract also increased CXCL8 secretion by primary canine sublingual epithelial cells [31]. Likewise, in canine epidermal keratinocytes, the *D. farinae* protein Der f 1 enhanced CXCL8 expression [10] and an *A. alternata* extract increased the secretion of this chemokine by human bronchial epithelial cells [51]. Although the composition of immune cells in the oral mucosa has not yet been studied in dogs, the oral cavity of humans and mice is known to contain low numbers of pro-inflammatory effector cells [1, 52] and the absence of pro-inflammatory signals likely facilitates the generation of antigen-specific tolerance. The induction of CXCL8 by certain allergens might therefore negatively impact the generation of allergen-specific tolerance.

The immortalised buccal epithelial cells’ CXCL8 secretion was also significantly increased when exposed to TLR1/2, TLR2, TLR2/6 and TLR5 ligands, but not by ligands for TLR3, TLR4, TLR7, TLR8 and TLR9. This corresponds for the most part with the findings of a study using human epidermal keratinocytes, which showed that these cells express TLR1, TLR2, TLR3, TLR5 and TLR6, while the cells’ expression of TLR4, TLR7 and TLR9 is questionable [25]. A CXCL8 response could also be induced by IL-17A, while IL-4, IL-6, IL-10, IL-17A, IFN-γ and TGF-β1 had no impact on the secretion of the chemokine. Similar to our results, IL-17A induced CXCL8 expression by human bronchial epithelial cells [53] and canine epidermal keratinocytes [22]. However, in another cell line of canine epidermal keratinocytes, IFN-γ suppressed CXCL8 expression while IL-17 had no significant effect [54]. IL-17A is a cytokine which can indirectly attract neutrophils to inflammatory sites. This cytokine’s expression was shown to correlate with a poor clinical outcome of allergen-specific sublingual immunotherapy in pollen-allergic children [55].

As a last group of substances, vitamin A and D metabolites were added to the buccal epithelial cell culture since these metabolites are also known to have immune modulating properties [56–59]. Interestingly, calcitriol could suppress CXCL8 secretion, a result which we had also observed in primary sublingual epithelial cells of the dog [31]. An anti-inflammatory role has been attributed to this vitamin D metabolite, increasing IL-10 secretion in dendritic cells and being able to induce regulatory T cells [60–62]. However, when primary human bronchial epithelial cells were exposed to calcitriol, CXCL8 expression was induced rather than suppressed [59]. The stimulatory properties of this metabolite might therefore be species-dependent and/or dependent on the epithelial cell type with which it interacts.

We studied cytokine production by canine buccal epithelial cells using a simplistic model where the cells were cultured as monolayers on culture plates. While these assays gave us insights on how the cells’ behaviour could be modulated by different stimuli, more accurate data could be obtained by culturing the cells in an environment which more closely mimics the natural conditions of the oral mucosa. Additionally, in vivo, oral epithelial cells are continuously exposed to bacteria of the oral microbiome [63]. Since we have shown that bacterial TLR ligands can impact canine buccal epithelial cells’ mediator secretion, it is reasonable to assume that, in vivo, these cells are in an altered immunologic state compared to the in vitro cultured immortalised buccal epithelial cells exposed to single stimuli as in our study [64].

In this study an immortalised buccal epithelial cell line was generated to get a better understanding of the repertoire of cytokines that is produced by the canine buccal epithelium in response to different stimuli. CXCL8 secretion was promoted by exposure to a *Dermatophagoides farinae* extract, an *Alternaria alternata* extract, the cytokine IL-17A and TLR1/2, TLR2, TLR2/6 and TLR5 ligands. Buccal epithelial cells therefore seem capable of generating a pro-inflammatory environment in the oral mucosa after exposure to different CXCL8-inducing stimuli. Calcitriol on the other hand suppressed CXCL8 production and could as such exert an anti-inflammatory function in these cells. The improved understanding of how substances influence cytokine secretion by buccal epithelial cells could provide an aid to immune modification strategies for vaccines administered within the oral cavity.

## Data Availability

The datasets used and/or analysed during the current study are available from the corresponding author on reasonable request.
